# Genetic Contribution of CISH Promoter Polymorphisms to Susceptibility to Tuberculosis in Chinese Children

**DOI:** 10.1371/journal.pone.0092020

**Published:** 2014-03-14

**Authors:** Lin Sun, Ya-qiong Jin, Chen Shen, Hui Qi, Ping Chu, Qing-qin Yin, Jie-qiong Li, Jian-ling Tian, Wei-wei Jiao, Jing Xiao, A-dong Shen

**Affiliations:** Key Laboratory of Major Diseases in Children and National Key Discipline of Pediatrics (Capital Medical University), Ministry of Education, Beijing Pediatric Research Institute, Beijing Children’s Hospital, Capital Medical University, Beijing, China; St. Petersburg Pasteur Institute, Russian Federation

## Abstract

Tuberculosis (TB) is the leading cause of death due to an infectious disease worldwide, particularly in developing countries. A series of candidate genes have been suggested to be associated with development of TB disease. Among them, the human Cytokine-inducible Src homology 2(SH2) domain protein (*CISH*) gene has been very recently reported to be involved in T cell activation and differentiation in response to *Mycobacterium tuberculosis* infection. Here, we studied the association between *CISH* promoter polymorphisms and pediatric TB. A case-control study enrolled 352 TB patients and 527 healthy controls, who were of Han Chinese ethnicity and aged from 0.2 to 18 years. *CISH* gene promoter SNPs rs414171, rs622502 and rs809451 were genotyped in all subjects and transcriptional activity, mRNA level, and plasma cytokine level of subjects with different genotypes were further examined. Carriers with rs414171TT homozygotes and rs809451GC heterozygotes had a 1.78-fold (95% CI,1.16–2.74) and 1.86-fold (95% CI, 1.26–2.74) excess risk of developing TB compared to those with wild-type genotypes. A greater risk of TB disease was observed in population carrying C_−809451_-T_−414171_-C_−622502_ haplotype (OR 3.66, 95% CI:2.12–6.32). The G_−809451_-A_−414171_-C_−622502_-containing *CISH* promoter drove a 5.43-fold increased reporter expression compared to the C_−809451_-T_−414171_-C_−622502_-containing counterpart in Hela cell lines (*P* = 0.0009). PBMCs carrying rs414171TT homozygotes and rs809451GC heterozygotes showed a reduced *CISH* mRNA level compared to cells carrying wild type genotypes. Individuals with the rs414171TT genotype had significantly increased IL-12p40 and IL-10 production. In conclusion, *CISH* promoter rs414171 and rs809451 polymorphisms may play a vital role in mediating individual susceptibility to tuberculosis.

## Introduction

Tuberculosis (TB) is the leading cause of morbidity and mortality worldwide, in particular, in Asia and Africa [1]. According to the fifth national tuberculosis epidemiological survey in 2010 in China [2], it was estimated that about 5 million new cases of pulmonary tuberculosis (PTB) occurred in China in 2010. It is known that the infection outcome is determined by the interaction between bacterial and host genetic components as well as by environmental, socio-economic, and nutrient factors. Candidate gene-association studies have successfully identified some of such host genes [3]–[6].

Cytokine-inducible Src homology 2(SH2) domain protein (*CISH*) gene was first associated with common infectious diseases in 2010 [7]. Human *CISH* is located on the third chromosome (3p21.3) with 4 exons spanning about 5kb and encoding a 258 amino acid polypeptide [8].

The human immune response to *Mycobacterium tuberculosis* (MTB) infection is a balance between inflammatory and anti-inflammatory responses in which cytokines play an important role. Members of the suppressor of cytokine signaling (SOCS) protein family were discovered as negative regulators of cytokine signaling by inhibition of the Janus kinase (JAK) - signal transducers and activators of the transcription (STAT) pathway [9]–[12], involved in the proliferation, differentiation, apoptosis and other immune regulation processes. *CISH* was the first described member of SOCS family [9] and was initially cloned as an immediate early gene that was induced by interleukin-2 (IL-2), interleukin-3(IL-3), and erythropoietin (EPO). In the process of inflammation, cytokines transmit the stimulating signal into cells through the JAK-STAT pathway, and regulate the transcription of specific genes including *CISH*. *CISH* is one of the STAT5-regulated genes: its expression is induced by STAT5 and it negatively modulates STAT5 activation by binding to cytokine receptors [13]-[15] and then inhibits the inflammatory response and immune regulation. Accumulating evidence supports the important regulatory role of *CISH* in T cell activation [16] and T cell differentiation in response to MTB infection in human [17].

These observations led us to investigate genetic variants in *CISH* for association with human susceptibility to TB. So far, only three articles reported the relationship between *CISH* polymorphisms and infectious diseases [7], [16], [18]. Khor et al. observed five single-nucleotide polymorphisms (SNPs) within the *CISH*-associated locus (at positions –639, –292, –163, +1320, and +3415) together in a multiple-SNP score, and found –292 (rs414171) accounting for most of the association signal (P = 4.58×10^−7^)[7]. The association of the above five SNP with risk of TB was studied in Han Chinese adult population, and AA genotypes of the SNPs rs2239751 and rs414171 were significantly associated with TB [16]. Finally, the vital role of rs414171A>T variant in hepatitis B virus infection was demonstrated in a Vietnamese population [18].

Children constitute 10–20% of the global burden of TB disease. With a prevalence of 91.8/100,000, Chinese pediatric TB contributes significantly to the global TB burden and more attention to pediatric TB is required to control worldwide tuberculosis. Pediatric TB patients have different clinical characteristics compared with adults. Extrapulmonary TB (EPTB) and severe TB are frequently diagnosed in children especially those younger than 1 year old [19]. In spite of this, little is known about gene polymorphisms and their possible role in the progression of different clinical types of TB in pediatric population.

Transcriptional regulation has been shown to be the major mechanism in regulating gene expression. The promoter region of *CISH* had been identified to cover several key regulatory elements, which may play a decisive role in the regulation of *CISH* transcription. Adult carriers of the rs414171 variant showed a muted *CISH* expression after stimulation by interleukin-2, as compared with wild-type cells [7]. Accordingly, we propose a hypothesis that *CISH* promoter polymorphisms may affect expression of the gene thus contributing to a substantial degree of inter-individual variability in the susceptibility to infectious diseases, including tuberculosis.

To the best of our knowledge, this is the first study of the *CISH* rs809451 polymorphism in the context of TB in Han Chinese pediatric population, whereas patients were stratified by the location and severity of the disease in order to clarify the role of *CISH* in pathogenesis of TB.

## Materials and Methods

### Study participants

A total of 352 patients were enrolled at the Beijing Children’s Hospital affiliated with Capital Medical University from July 2004 to May 2010. All TB cases were classified according to the diagnostic standards of the American Thoracic Society (ATS) [20], the Pediatric TB clinical diagnosis standard in China [21] and WHO guidelines for disease severity classification for non-HIV related TB [22].The diagnosis of pediatric TB was based on (i) etiology or pathology results (Acid-Fast Bacilli Stain, culture, fibreoptic bronchoscopy observation), (ii) clinical signs (typical symptoms and imaging evidences, close contact with known active TB case, tuberculin skin test positive results), (iii) positive clinical response to anti-TB therapy, (iv) exclusion of other lung disease, such as, pneumonia, lung tumor etc. Clinical TB was diagnosed if positive features of (i) OR (ii) plus (iii) OR (ii) plus (iv) were present.

In the present study, pediatric TB patients were diagnosed to be pulmonary TB (PTB, pathological changes limited to lung) or extrapulmonary TB (EPTB, pathological changes involving lung and other tissues). Severe tuberculosis (SevTB) included patients with two or more noncontiguous sites, miliary mottling, or involvement of meningitis, pericardium, spinal, intestinal and splenic sites with or without lung involvement. None of the patients had a clinical history of diabetes mellitus, HIV infection, or received an immunosuppressive therapy.

The control group included 527 children who admitted to the hospital for annual physical examination, without history of TB, without MTB infection, with normal radiographic examination findings, and negative tuberculin skin test results at the enrollment. Control individuals were matched to TB patients by age, sex, and ethnicity. Children with hereditary diseases and immunodeficiency and incomplete clinical data were excluded. All controls were followed for 2 years after their initial visit, to ensure that they had no latent TB infection at the time of the study. All study individuals were of the Chinese Han ethnicity.

The case group (n = 352) included 148 (42.0%) cases of active pulmonary TB (PTB), 204 (58.0%) cases of extra-pulmonary TB (EPTB). Severe tuberculosis (SevTB) was identified in 169 (48.0%) patients. The mean age was 6.0 years (SD 4.7, range 2 months-16.5 years) for patients and 6.6 years (SD 4.0, range 3 months-18 years) for controls. All study subjects were BCG vaccinated at birth. The demographic characteristics and diagnostic modality used for confirmation of TB are explained in Table 1

**Table 1:** Demographic characteristics of study population.

Characteristic	TB patients				Total TB	Controls
	PTB(n = 145)	EPTB(n = 207)	SevTB(n = 166)	non SevTB(n = 166)	n = 352	n = 527
Gender, Male/Female	89/56	135/72	108/58	116/70	224/128	380/147
Age, Mean years (SD)	6.4(4.7)	5.2 (4.6)	5.1 (4.6)	6.5(4.8)	6.0(4.7)	6.6(4.0)
TST positive (n)	132	179	140	171	311	0
TSPOT-TB test positive (n)	19	22	8	33	41	0
Acid-Fast Bacilli Stain positive (n)	8	12	9	11	20	0
Culture positive (n)	13	9	4	17	21	0
Imaging change (n)	148	119	102	165	267	0
FOB Observation	51	17	12	56	68	0

PTB,pulmonarytuberculosis;EPTB,extra-pulmonaryTB; SevTB,severe tuberculosis; TST, tuberculosis skin test; FOB, fiberoptic bronchoscopy.

Clinical investigation was conducted according to the principles expressed in the Declaration of Helsinki. This research was approved by the Ethics Committee of Beijing Children’s Hospital. Written informed consent was obtained from the patients or the guardians of patients that participated in this research..

### SNP selection and DNA analysis

According to CHB data at public HapMap database (http://hapmap.ncbi.nlm.nih.gov), minor allele frequencies (MAF) of three *CISH* SNPs in Chinese Han population were greater than 0.05 (including rs414171, rs622502 and rs809451); these SNPs were retained for further genotyping. *CISH* gene was amplified by PCR. The primers used are listed in Table 2.PCR products were sequenced using a 3730 DNA Analyzer (Applied Biosystems, Foster City, CA, USA).

**Table 2:** Primers used in PCR experiment.

Method	Aim	Primers
PCR	Genotyping of rs414171 and rs622502	F: agttccaccgcgagataagag
		R: ggaagcagcgtcttcctaga
	Genotyping of rs809451	F: ttgtaactctgttgcctggc
		R: cgggcttcatgagtgcaac
Real time PCR	Expression of *CISH*	F:ctgtgcatagccaagaccttctc
		R: ctggcatctgcaggtgtt
	Expression of control gene (human beta-2 microglobulin)	F: tgacaacgaatttggctaca
		R: ggggtctacatggcaactg

### Plasmid constructs

Two luciferase reporter gene constructs were generated by PCR spanning –1883 to –62 bp of the *CISH* promoter region and containing C-809451-T-414171-C-622502 or G-809451-A-414171-C-622502 haplotypes. Promoter genotypes were independently inserted between the NheI and XhoI restriction sites of the pGL3-basic plasmid (Promega, Madison, WI, USA) to generate the pGL3-CISH plasmids. All constructs were sequenced to confirm the allele, orientation and integrity of each insert.

### Cell culture and luciferase assays

HeLa cells were cultured in Dulbecco’s modified Eagle’s medium (Gibco, BRL, USA) supplemented with 10% fetal bovine serum, penicillin (100 U/ml), and streptomycin (100 U/ml) and incubated at 37°C in a 5% CO_2_ atmosphere. Constructed pGL3-*CISH* plasmids were transfected into HeLa cells with the pRL-SV40 plasmid (Promega, USA) by using Lipofectamine 2000 (Invitrogen, Carlsbad, CA, USA). Firefly luciferase and renilla luciferase (both via pRL-SV40 plasmid) activities were sequentially measured 48 h after transfection by a luminometer, utilizing a Dual-Luciferase reporter assay system (Promega, USA). Results were expressed as relative light units of firefly luciferase activity over relative light units of renilla luciferase activity. All experiments were performed in triplicate and repeated three times.

### Expression levels of CISH in individuals with different genotypes

Peripheral blood mononuclear cells (PBMCs) were separated by ficoll gradient density centrifugation method. Cells were resuspended in RPMI medium with 10% fetal bovine serum, seeded into each well of a 24-well plate (2×10^6^cells/ml), incubated in 5% CO_2_ at 37°C and stimulated with IL-2 (final concentration of 100 U/ml). Cells were harvested at 0h, 1h and 2h after addition of IL-2 and total RNA was extracted and used for cDNA synthesis. Real-time PCR was performed in an ABI7300 Sequence Detection System (Applied Biosystems, USA) using SYBRPremix Ex Taq II (TaKaRa Bio, Japan). The primers used are listed in Table 2.

### Plasma cytokine study

The cytokine assays were prepared by diluting venous blood 1:5 with RPMI 1640 (Invitrogen, USA), plating it in a 96-well dish, stimulating by inactivated *M. tuberculosis* H37Rv (the concentration of bacteria is 7.5×10^3 ^μL) for 20 hours, and then harvesting supernatants. The concentration of cytokines in cell culture supernatant samples was quantified using a customized Milliplex MAP Human Cytokine/Chemokine Panel (# HCYTOMAG-60K, Millipore, Boston, MA, USA). The assay was performed according to the manufacturer’s instructions. Standards and samples were analyzed in duplicates on a Luminex 200 device (Luminex, Austin, TX, USA) using the MilliplexTM Analyst Software (Version 3.5, Millipore, USA).

### Genetic and Statistical analysis

Statistical analysis was carried out using the Statistical Package for SNPStats software (http://bioinfo.iconcologia.net/snpstats/start.htm) and SAS version 9.1 (SAS Institute, Cary, NC). Haplotype analyses were performed using the SHEsis program (http://analysis.bio-x.cn/myAnalysis.php). Haplotype frequencies were inferred using the derived expectation maximization (EM) algorithm as implemented in the SHEsis software. Adjusted odds ratio (AOR) and 95% confidence interval (CI) were calculated by logistic regression analysis. Differences between non-contiguous variables, genotype distribution and allele frequency were tested by Fisher’s exact test. Univariate analysis was performed for categorical variables with a χ2 test and for continuous variables using a Mann-Whitney U test with 2-sided testing. T-test was used to compare the promoter activity between haplotypes. An allelic P value of less than 0.05 was considered nominally significant.

## Results

### Genotyping and genetic analysis

Three SNPs in *CISH* promoter region with minor allele frequency (MAF) value above 0.05 were selected for this study. The genotyping results of *CISH* are shown in Table 3. All SNPs were in HWE in both control group and TB group. Rs414171T, and rs809451C allele showed a significant increase in TB group (P = 0.016 and 0.002 respectively), and indicated an association with an increased risk of developing TB, with AORs of 1.27 (95% CI, 1.05–1.55) and 1.73 (95% CI, 1.22–2.46), respectively. We then analyzed the differences between TB patients and controls in the distribution of these genotypes and their association with risk of TB. Rs414171TT homozygote was found in 15.9% of patients compared with 11.0% in controls (AOR = 1.78, 95% CI, 1.16–2.74; P = 0.022). Rs809451GC heterozygote was found in 18.8% of patients compared with 10.8% in controls (AOR = 1.86, 95% CI, 1.26–2.74). Genotypes distribution of the rs622502 C/G polymorphism was not significantly different between patients and controls.

**Table 3:** Relationship between *CISH* gene promoter polymorphisms and pediatric TB.

Site/Genotype/allele	Controls, n = 527, n(%)	TB patients, n = 352, n(%)	OR^a^(95% CI)	P^a^
**rs414171 A/T**				
AA	228(43.3)	130(36.9)	Reference	
AT	241(45.7)	166(47.2)	1.24(0.93–1.67)	0.641
TT	58(11.0)	56(15.9)	1.78(1.16–2.74)	0.022
AA+ AT	469(89.0)	296(84.1)	Reference	
TT	58(11.0)	56(15.9)	1.58(1.06–2.36)	0.025
A	697(66.1)	426(60.5)	Reference	
T	357(33.9)	278(39.5)	1.27(1.05–1.55)	0.016
**rs809451 G/C**				
GG	466(88.4)	283(80.4)	Reference	
GC	57(10.8)	66(18.8)	1.86(1.26–2.74)	0.279
CC	4(0.8)	3(0.9)	1.36(0.30–6.27)	0.998
CC+GC	61(11.6)	69(19.7)	1.83(1.25–2.67)	0.002
G	989(93.8)	632(89.8)	Reference	
C	65(6.2)	72(10.2)	1.73(1.22–2.46)	0.002
**rs622502 C/G**				
CC	446(84.6)	307(87.2)	Reference	
CG	80(15.2)	45(12.8)	0.72(0.55–1.21)	0.325
GG	1(0.2)	0(0)	-	0.432
GG+CG	81(15.4)	45(12.8)	0.80(0.57–1.25)	0.324
C	972(92.2)	659(93.6)	Reference	
G	82(7.8)	45(6.4)	0.81(0.56–1.18)	0.271

ORs were adjusted for sex, age, in a logistic regression model.

We further examined association of rs414171A/T and rs809451G/C polymorphism genotypes with different clinical forms of TB (Table 4). For rs809451G/C polymorphism, either in PTB or EPTB, non SevTB or SevTB, the risk was significantly higher in individuals with the C-allele (CC+GC) than those without it. In TB subgroups, statistical difference of rs414171A/T and rs809451G/C polymorphism was not found between PTB vs EPTB and non SevTB vs SevTB by using logistic regression (Table 4).

**Table 4:** Relationship between *CISH* promoter polymorphism and pediatric tuberculosis (TB), stratified by location and severity of the disease.

genotype	Patients (n)		Controls (n)		a OR(95%CI)	a *P*
	1	2	1	2		
rs414171 A/T						
location						
PTB	122	23	469	58	1.58(0.93–2.68)^3^	0.089
EPTB	174	33	469	58	1.58(0.99–2.52)^3^	0.055
					1.05(0.63–2.28)^4^	0.873
severity						
non SevTB	157	29	469	58	1.54(0.95–2.50)^3^	0.082
SevTB	139	27	469	58	1.64(0.99–2.71)^3^	0.055
					0.90(0.50–1.60)^4^	0.716
rs809451G/C						
location						
PTB	32	113	61	466	2.14(1.33–3.44)^3^	0.002
EPTB	37	170	61	466	1.61(1.03–2.53)^3^	0.037
					0.75(0.44–1.28)^4^	0.294
severity						
non SevTB	37	149	61	466	1.88(1.20–2.95)^3^	0.006
SevTB	32	134	61	466	1.77(1.10–2.85)^3^	0.019
					1.07(0.63–1.82)^4^	0.813

1: AA+ AT for rs414171, CC+GC for rs809451; 2: TT for rs414171, GG for rs809451.

ORs were adjusted for sex, age, in a logistic regression model.

3: OR and *P* value was calculated between patients and controls, 4: OR and *P* value was calculated between subgroups of TB (PTB vs EPTB, non SevTB vs SevTB).

### Haplotypes and risk of TB

Haplotype analysis was performed for these three polymorphisms. Accepting the lowest frequency threshold for this haplotype analysis to be 0.05, three haplotypes were identified (Table 5). Population carrying two risk alleles (C_−809451_-T_−414171_-C_−622502_) had increasing odds of disease susceptibility (OR 3.66, 95% CI:2.12–6.32; P = 7.53×10^−7^). On the contrary, G_−809451_-A_−414171_-C_−622502_ haplotype showed a significantly decreased TB risk (OR 0.76, 95% CI:0.62–0.94; P = 0.009).

**Table 5:** *CISH* promoter haplotypes distribution in tuberculosis patients and healthy control subjects.

Haplotypes^1^	Patients, n(%)	Control, n(%)	?2	*P*	OR(95% CI)
G_−809451_-A_−414171_-C_−622502_	413(58.6)	670(63.6)	6.76	0.009	0.76 (0.62–0.94)
G_−809451_-T_−414171_-C_−622502_	219(31.1)	308(29.3)	0.41	0.525	1.07 (0.87–1.32)
C_−809451_-T_−414171_-C_−622502_	45(6.4)	19(1.8)	24.51	7.53*10^−7^	3.66 (2.12–6.32)

1 Haplotypes were estimated with the expectation maximization algorithm.

ORs were adjusted for sex, age, in a logistic regression model.

### Promoter activity of *CISH*

**Figure 1:**
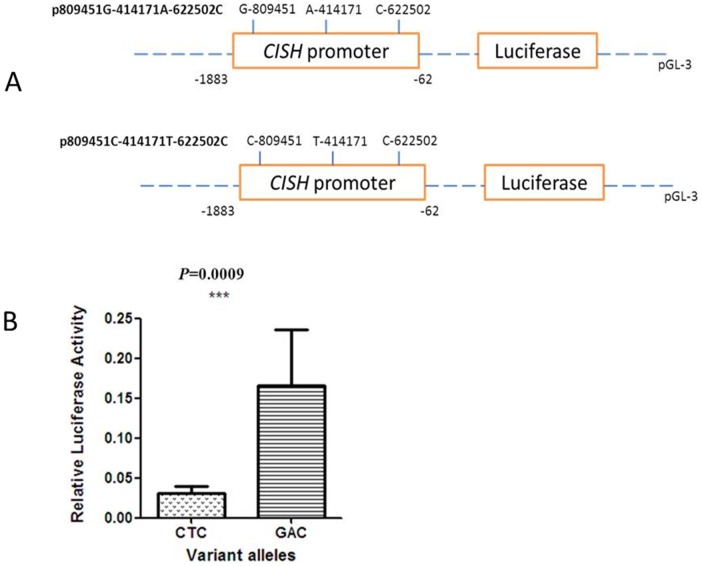
Transient reporter gene expression assays with constructs containing *CISH* promoter. (a) Scheme of the reporter gene constructs with *CISH* promoter differing in rs809451 C>G and rs414171 A>T sites. (b) Luciferase expression of the two constructs in Hela cells cotransfected with pRLSV40 to standardize transfection efficiency.

We constructed a promoter region containing rs414171 and rs809451 alleles in pGL3-basic plasmid to generate pGL3-*CISH* plasmids for analysis of the transcriptional activity of different promoters. As shown in Figure 1, the G_−809451_-A_−414171_-C_−622502_-containing *CISH* promoter drove a 5.43-fold increased reporter expression in Hela cell lines compared to the C_−809451_-T_−414171_-C_−622502_-containing counterpart (*P* = 0.0009).

### Expression levels of *CISH* in lymphocytes with different genotype

**Figure 2:**
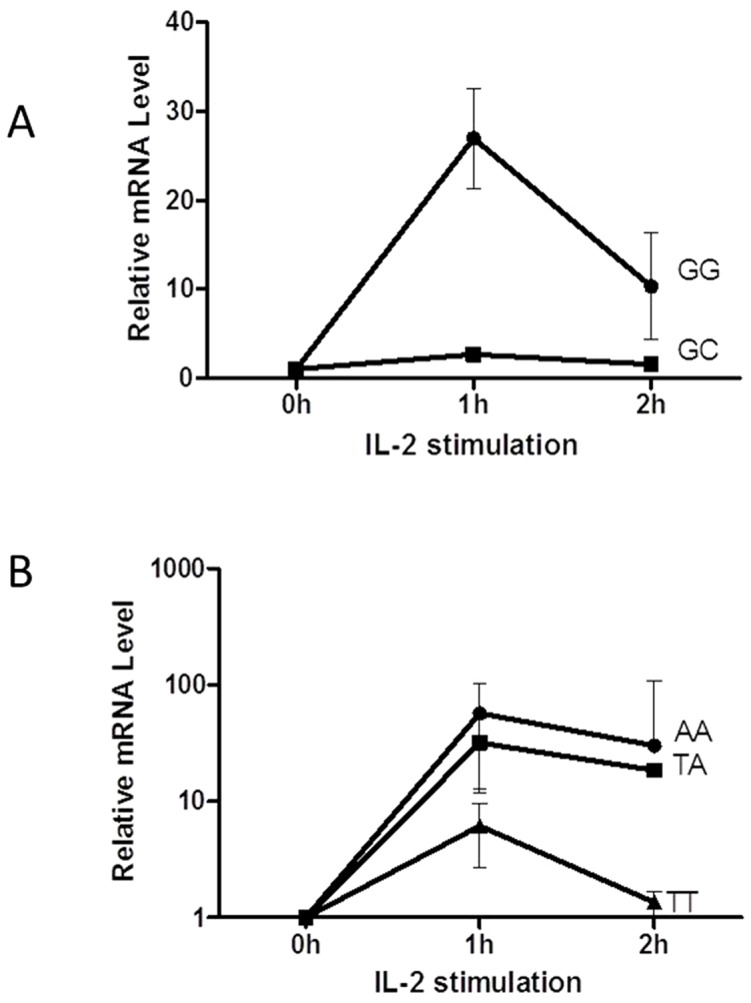
*CISH* mRNA expression level in PBMC as function of *CISH* genotype. (a) Expression level of the rs809451GG genotype was significantly higher than that of the GC genotype (p < 0.05). (b) Expression level of the rs414171AA or AT genotype was significantly higher than that of the TT genotype (p < 0.05).

Because of the low frequency of rs809451CC in controls (0.8%), we screened 200 healthy children, but found none carrying this genotype. The effect of the rs809451G/C SNP on *CISH* expression was only compared between carriers with GG and GC genotypes. It was found that lymphocytes carrying the rs809451 GC genotype had significantly decreased *CISH* expression level compared to the cells carrying GG genotype. Subjects with the rs414171 TT genotype had significantly lower mRNA expression level than those with AA and AT genotypes (Fig. 2).

### Plasma cytokine levels of subjects with different genotype

**Figure 3:**
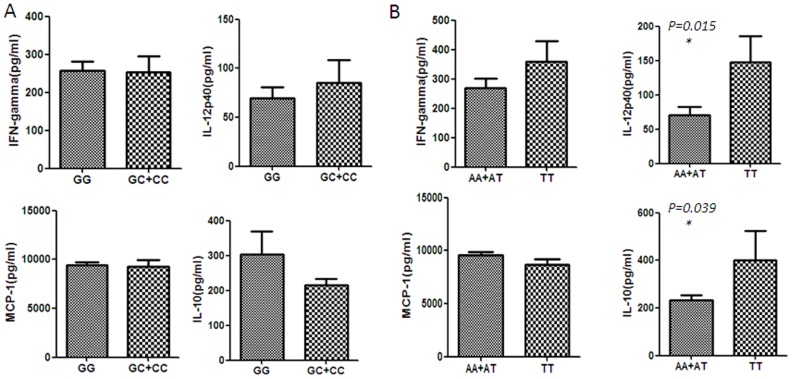
Serum levels of IFN-gamma, IL12p40, MCP-1, IL-10 in individuals with different *CISH* genotypes. (a) rs809451GG versus rs809451GC/CC genotypes. (b) rs414171AA/AT versus rs414171TT genotypes.

We collected 68 whole blood samples from the controls with different rs414171 and rs809451 genotypes and examined cytokine production using an ex vivo assay (Fig. 3a and 3b). There was an apparent trend of the significantly increased IL-12p40 and IL-10 production in samples from individuals with rs414171 TT genotype compared to carriers of the AA+AT genotypes.

## Discussion

The CISH proteins inhibit cytokine signaling through various mechanisms, affecting the signal mediator function and inhibiting the interaction between cytokine receptor and signaling mediator [23]–[25]. Expression of CISH strongly promoted T cell proliferation, cytokine production, and prolonged survival of activated T cells. The bidirectional regulation of T cell activation and cytokine signaling by CISH represents an important mechanism for controlling both immune responses and homeostasis [26]. A previous study has found that the susceptibility to some infectious diseases is associated with a *CISH* polymorphism [27] which indeed may affect the capacity of T cells to respond to inflammation caused by a pathogen, e.g. MTB.

Exposure to MTB can result in numerous different clinical outcomes. In case of mutation, different immune response genes may be responsible for different clinical presentations. Little is known about *CISH* polymorphisms and their possible relationship to active TB with different outcomes.

Compared with previous studies [7], [16], this study was distinctive. First, it targeted a pediatric population. Childhood TB is known to be commonly extra-pulmonary, disseminated and severe, and is associated with high morbidity and mortality [28]. Second, instead of examining five SNPs spanning the whole gene, this study mainly focused on the promoter region, and investigated the rs809451 polymorphism in the context of TB susceptibility for the first time. Third, we studied the mechanism of the potential relation between these polymorphisms and the risk of TB.

We found a positive association of rs414171T and rs809451C alleles with TB disease. Under the recessive model, persons with rs414171 TT genotype were 1.58-fold more likely to develop TB than those with AA and AT genotypes. Patients with the CC and GC genotype were 1.83-fold more likely to develop TB than those with the GG genotypes. C-_809451_-T-_414171_-C-_622502_ haplotype that include two TB risk alleles, was in higher rate in TB group compared to the control group (OR 3.66, 95% CI:2.12–6.32; P<0.000).

We found a positive association between the rs414171 TT genotype and EPTB and SevTB. The rs809451 C allele was linked with the increased risk of TB in the dominant model in each subgroup. However, statistical differences of allelic and genotypic distributions were not observed between TB subgroups in our study (PTB vs. EPTB, or nonSevTB vs. SevTB).

We hypothesized that the TB-susceptible rs414171 T and rs809451 C alleles could lead to reduced *CISH* transcription and tested this hypothesis by dual-luciferase reporter analysis. As a result, the transcriptional activity of the C-_809451_-T-_414171_-C-_622502_ haplotype promoter was significantly lower than that of the G_809451_-A_−414171_-C_−622502_ allele promoter. This result is consistent with our data of RNA expression in subjects with different genotypes: the lowest expression of RNA correlated with TB-susceptible rs809451 GC and rs414171 TT genotypes. Khor et al. [7] also demonstrated the decreased transcriptional activity of rs414171 T allele after stimulating cells with IL-2.

In addition, stimulation of PBMCs from control persons carrying the TT genotype with *M. tuberculosis* antigens yielded higher concentrations of IL-12p40 and IP-10, compared with those carrying the AA+AT genotypes. Th1 cytokines is important for TB control in humans. IL-12p40 is a component of Th1 cytokine IL-12 and IL-23 and is required for their binding to the IL-12 receptor 1 subunit [29]. IL-12p40 homodimers function as a macrophage chemo-attractant as well as a competitive antagonist of IL-12 [30]–[32]. Previous study has confirmed that increasing IL-12p40 production is a sputum biomarker of AFB positive TB, and likely reflects less effective immune control of TB [33]. IL-10 is a pleiotropic anti-inflammatory cytokine that is produced by immune cells and indirectly regulates cellular recruitment to the site of infection [34]–[36]. IL-10 works in concert with other regulatory mechanisms, in order to suppress cellular function, e.g. down regulates activated macrophages [37], [38].

The findings thus highlight that persons bearing *CISH* rs414171 TT and rs809451 CC genotypes are at increased risk for progression of TB infection to active disease, with an underlying mechanism related to the decreased promoter activity, mRNA expression level and higher concentrations of IL-12p40 and IL-10. In addition, we predict that rs414171 and rs809451 are located on binding sites of specific transcription factor using Gene Regulation software (http://www.gene-regulation.com/pub/programs.html). The *CISH* promoter with rs414171 T allele showed a stronger binding ability to transcription factor specificity protein 1(SP1), and *CISH* promoter with TB-susceptible rs809451C allele had stronger binding ability to SP1 and T3R-alpha. However, further studies are needed to confirm these computer predictions.

In conclusion, the major findings of this study of *CISH* gene are as follows: (i) both the rs414171 TT genotype and rs809451 CC genotype act as possible risk factors for clinical TB; (ii) transcriptional activity of the C-_809451_-T-_414171_-C-_622502_ haplotype promoter is weaker than that of the G-_809451_-A-_414171_-C-_622502_ haplotype promoter; (iii) carriage of the mutant rs414171 TT and rs809451 GC genotypes resulted in markedly lower gene expression in response to interleukin-2 stimulation, compared with wild-type genotypes; (iv) correlation of *CISH* genotypes and cytokine levels suggests that persons with the rs414171 TT genotype have higher level of IL-12p40 and IL-10 production, which may suppress effective immune control of TB. The findings of the present study will contribute to our advance in understanding the molecular mechanisms underlying the progression of TB disease. Additional studies in other populations are warranted to test our findings.
